# Concentration-Dependent Emission of Annealed Sol-Gel Layers Incorporated with Rhodamine 19 and 6G as the Route to Tunable High-Temperature Luminescent Materials

**DOI:** 10.3390/gels8070408

**Published:** 2022-06-28

**Authors:** Maria Zdończyk, Bartłomiej Potaniec, Marta Fiedot-Toboła, Tomasz Baraniecki, Joanna Cybińska

**Affiliations:** 1 Faculty of Chemistry, University of Wrocław, F. Joliot-Curie 14 Street, 50-383 Wrocław, Poland; maria.zdonczyk@chem.uni.wroc.pl; 2 Advanced Materials Synthesis Group, Łukasiewicz Research Network—PORT Polish Center for Technology Development, Stabłowicka 147 Street, 54-066 Wrocław, Poland; bartlomiej.potaniec@port.lukasiewicz.gov.pl (B.P.); marta.fiedot-tobola@port.lukasiewicz.gov.pl (M.F.-T.); tomasz.baraniecki@port.lukasiewicz.gov.pl (T.B.)

**Keywords:** sol-gel, organic dyes, luminescence, tunable emission, rhodamine

## Abstract

The sol-gel technology allows for the development of materials for nonlinear optics and photonics through the synthesis of multifunctional ceramic materials. Although the nature of the amorphous matrix allows the material to be doped with a large amount of the active components without quenching, it may affect the spectroscopic characteristics of the dye (e.g., result in a shift of absorption and emission peaks with drying time, presumably with a change of concentration). This study presents the material (SiO_2_ impregnated with organic dyes—Rhodamine 6G and 19) with tunable emissions obtained by the authors upon annealing at different temperatures within the range of 100–300 °C. Possible observed effects were discussed based on spectroscopic properties and thermal studies of the synthesized material. Concerning annealing at different temperatures, an effect on concentration was observed. At the same time, a longer heating process at 300 °C revealed a protective function of sol-gel-derived silica for the organic dye; the longer heating did not cause any further significant changes in the dye’s emission, which indicates the preservative role of the sol-gel layers. Furthermore, etching tests of thin layers were conducted, resulting in smooth side edges of the waveguide. The tests have shown that it is possible to use dye-doped sol-gel layers as active components in photonics platforms.

## 1. Introduction

Experiments with dyes and sol-gel methods began in 1990 [1,2,3]. The sol-gel technology allows for the development of materials for nonlinear optics and photonics by synthesizing multifunctional ceramic materials in the form of xerogels. The reactions are often carried out at room temperature, enabling the stable incorporation of organic dyes without losing their spectroscopic properties. These materials are used in lasers [4], solar panels [5], numerous optical sensors [6,7,8], and drug delivery [9]. Despite the exceptional spectroscopic properties of organic dyes, the sol-gel technology faces problems not observed in doping with inorganic compounds. The nature of the amorphous matrix, although it allows the material to be doped with a large amount of the active ingredient without quenching [10], may affect the spectroscopic characteristics of the dye (e.g., result in a shift of absorption and emission peaks with drying time, presumably with a change in concentration [11,12]). The term “doping” could be used in the case of a porous xerogel matrix with dye particles [13,14]. However, in the case of conventional glasses, the term “impregnated with dye” is also used [12].

The rhodamines used in this study are well-known fluorescent dyes with high quantum yields. A previous study on their behavior in silica host crystal matrices shows that they can exist in the form of isolated molecules or aggregates called H- or J-dimers [15]. H-dimers do not exhibit luminescent properties. Instead, their occurrence can be determined based on absorption properties—an absorption band characterizes a non-fluorescent H-dimer shifted towards higher energies (H-band). In contrast, J-dimers are described by an emission band shifted towards lower energies [16]. Previous research on sol-gel hybrids with rhodamine concerned nanoparticles and films doped with Rhodamine B [14,17,18,19,20], Rhodamine 110 [14,21,22,23,24], Rhodamine 101 [21,25,26], Rhodamine 6G (Rh6G) [14,27,28,29,30], Rhodamine 19 (Rh19) [11,13], and Rhodamine 800 [31,32,33,34], with observations on how the spectroscopic properties of the dyes allow them to be used in sol-gel matrices for the applications mentioned above.

The search for high-temperature (degradation temperature above 300 °C) dyes for potential applications in photonics led the authors to use xanthene dyes in materials with thermal stability up to 300 °C (e.g., Rhodamine 110, with a degradation temperature over 400 °C). However, new xanthene derivatives, synthesized as part of our earlier study, did not show emission after the annealing process [35,36].

Rhodamines have well-known spectroscopic properties, which allow us to determine the properties of materials doped with them. Rhodamine 19 and Rhodamine 6G, with their structural difference in the form of an anion present [37], were chosen from the group, leading to their comparison in one study. It is worth emphasizing that previous research on using rhodamines in sol-gels for hybrid photonics platforms focused on testing them below the temperature of 275 °C. One study described the GAPDV method [38], and another described the wet sol-gel method [10]. What the two works have in common is that the silica used is porous. However, in our work, we do not use surfactants that constitute an organic template for the pores. The use of non-porous silica made it possible to obtain stable layers above 300 °C. This study focuses on different spectroscopic properties after annealing at higher temperatures. The possible effects observed in the final materials were analyzed by employing temperature studies and experiments on dye solutions. Furthermore, an etching test of a thin sol-gel layer applied to a glass substrate was performed. This issue is crucial as it shows that it is possible to use such components as active layers in photonics technologies requiring high temperatures while maintaining luminescent properties.

## 2. Results and Discussion

According to the procedure described in Supplementary Materials (Section S1), samples were prepared and then subjected to spectroscopic (emission and absorption spectra) and temperature tests in accordance with the experimental section shown in the Supplementary Materials (Section S2). Xanthene dyes dissolve well in the solvent used in sol-gel synthesis—ethanol. Therefore, it was possible to prepare a homogeneous sol. Per literature reports, homogeneity of the sol is crucial for obtaining a gel with good mechanical properties. During the annealing process, the color of the samples and the emission color changed. Considering the possible effects of such behavior, the authors focused on two issues:

(1) Effects in the immediate environment of the organic dye (shift of the emission maximum with a change in concentration or change in pH, organic dye decomposition during the heating process);
(2) Preservation of the dye in the silica parent matrix.

### 2.1. Effect in the Immediate Environment of the Organic Dye (Spectroscopic Study)

**Figure 1:**
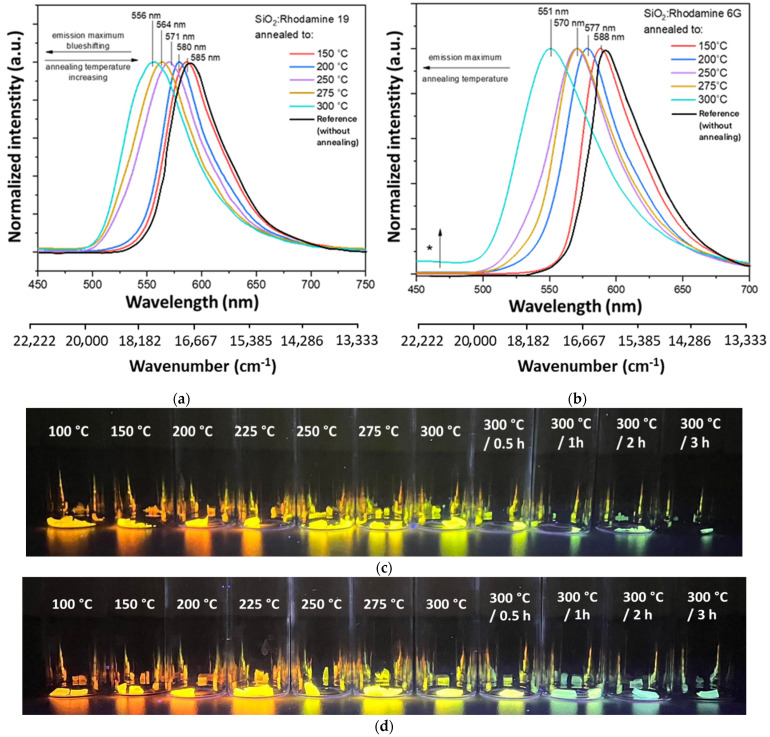
Normalized emission spectra for gels annealed from 150 °C to 300 °C for 10 min, λ_ex_ = 280 nm. Marked with an asterisk−an additional band associated with emission from silica matrix: (**a**) SiO_2_: Rhodamine 19; (**b**) SiO_2_: Rhodamine 6G. Photograph of samples upon excitation with UV lamp λ_ex_ = 360 nm impregnated with dye: (**c**) Rhodamine 19; (**d**) Rhodamine 6G.

As shown in Figure 1a, material doped with Rhodamine 19 shows a substantial blue shift (shift of emission maxima towards higher energies) with increasing annealing temperature. The shift is about 30 nm (892 cm^−1^), from 585 nm observed at 150 °C to 556 nm at 300 °C. Photographs of annealed layers (Figure 1c) show different emission colors depending on the applied annealing temperature upon excitation with a UV lamp (λ_ex_ = 360 nm).

Analogous observations were made for samples doped with Rhodamine 6G (Figure 1b,d). The shift in the case of these materials is higher—around 37 nm (1142 cm^−1^). The results obtained for layers annealed at temperatures above 250 °C are consistent with literature reports on low-temperature layers doped with Rhodamine 19 [11,13] and 6G [14,25,26]. It is noticeable that the shape remains similar even if the band position changes. However, at 300 °C, an additional broad band is observed (marked with the asterisk in Figure 1b) at about 450 nm, most likely attributed to the emission from SiO_2_ [39,40]. The chromaticity diagram (Figure S2) shows changes in emission color from reddish to greenish, which suggests the possibility of obtaining an emitter of different colors starting from the same parent dye used.

Absorption spectra for synthesized materials are shown in Supplementary Materials (Figure S3). Rhodamine 19-doped gel absorption maxima showed a blue shift with increasing heating temperature from 535 nm to 510 nm. The literature shows that the absorption maximum is placed at around 520 nm in methanol [10,11]. For Rhodamine 6G-doped layers, it is noticeable that spectra change upon heating to 200–250 °C. First, absorption spectra contain two main peaks (495 nm and 530 nm), while the second has a major peak at 505 nm. The observed blue shift is consistent with available literature data [13]. However, in the case of materials obtained in the study, absorption at 505 nm is observed even upon heating to 300 °C.

It should be stated that, upon gelation, samples started to decrease in volume due to the drying process. During gelation, pH rises, which indicates changes in spectroscopic properties. To check how a change in pH affects the spectroscopic properties of both rhodamines, measurements were performed in a variable pH of the solvent and a variable concentration of the sample. For Rh19 solutions, two different pH dependencies were observed (Figure S4) for acidic or strongly alkaline solutions at 565 nm, while for slightly alkaline solutions, this was observed at 555 nm. For Rh6G solutions, the shift of emission maxima towards lower wavelengths was visible without any pH changes. Results for organic dye solutions are consistent with literature reports for analogous compounds [41,42].

**Figure 2:**
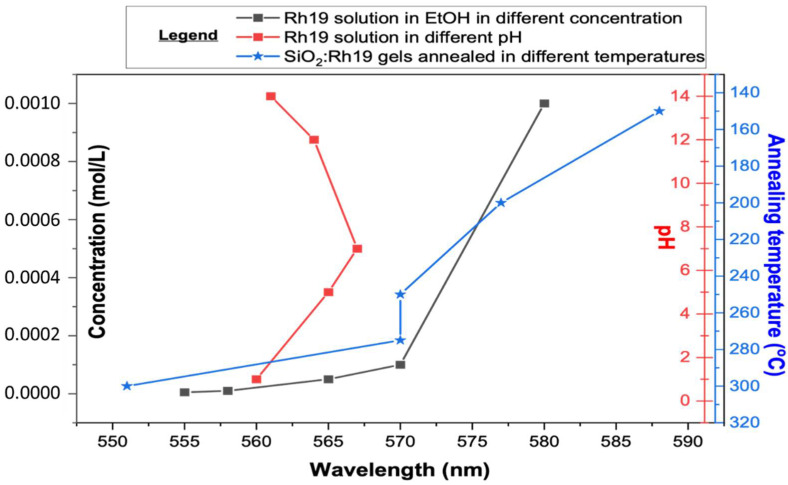
The relationship between pH and concentration compared to the results obtained with the gels for Rhodamine 19 (Rh 19)-doped materials (abbreviations used: EtOH—ethanol).

As the pH-dependent effect did not explain the significant shift towards high energies, concentration-dependent measurements were also made. On their basis, a spectroscopic effect similar to the synthesized material became visible. High-concentration Rhodamine 6G solution emission can be observed above 600 nm in the range of 600–680 nm [43]. With decreasing dye content, a blue shift of luminescence is observed. It appears that intensity also changes, reaching a maximum at 10^−4^ g/mL Rh6G. The blue shift begins to even out with further dilution, and the intensity drops again [44]. It was observed that for intermediate concentrations, the shape of the spectrum indicates two overlapping components [45]. The relationship between pH and concentration compared to the results obtained with the gels is plotted in Figure 2 (while measured spectra are shown in Supplementary Figure S5).

As a result of the performed spectroscopic measurements and based on literature reports, it was concluded that the concentration-dependent emission effect is observed in materials annealed up to 300 °C. Dilution of the material is caused by annealing since some of the dye is expected to be absorbed onto the material’s surface. At the same time, some dye particles are trapped in an amorphous silica lattice. By comparing the measurements made for the solutions by authors and the literature reports, it was possible to infer the amount of dye retained in the final material (Table 1). The comparison shows that we can determine dye concentration in the silica matrix remaining upon the annealing process.

### 2.2. Preservation of the Dye in the Silica Matrix (Spectroscopic and Thermal Study)

**Figure 3:**
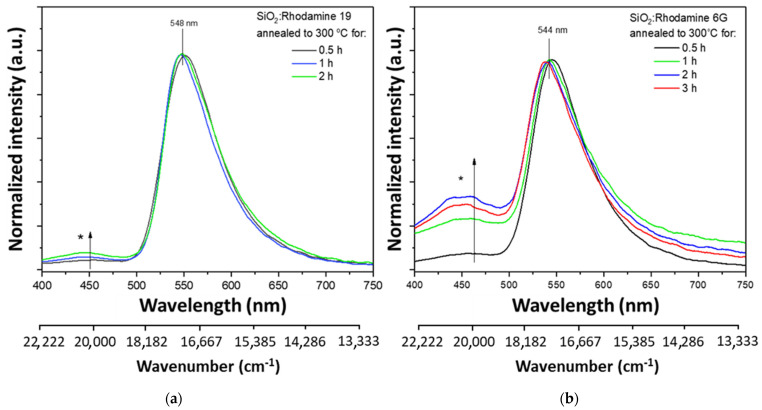
Normalized emission spectra for gels annealed at 300 °C for different time periods, λ_ex_ = 280 nm. Marked with an asterisk−an additional band associated with emission from silica matrix. As the annealing time is lengthened, the ratio of the dye-derived band to the silica changes in favor of silica: (**a**) SiO_2_: Rhodamine 19; (**b**) SiO_2_: Rhodamine 6G.

In order to test the protective properties of the dried gels, the sol-gel-derived materials were heated at 300 °C for 0.5–3 h. Earlier in the study, it was concluded that dye particles were also trapped in the sol-gel matrix. In both materials, the intensity ratio between the organic dye band and the additional one changes (Figure 3). A more outlined spectrum is observed in the case of the Rhodamine 6G-doped material. In absorption spectra (Figure S3) in both materials upon annealing at 300 °C for different time periods, a decrease in absorbance can be observed, although the maximum is placed in the same wavelength—505 nm. Additionally, a higher absorbance is visible in the UV region.

However, the maxima derived from organic dyes do not shift in either case [39]. In the matter of sol-gel materials, the optical properties are mainly determined by the luminescence centers located on the surface, as their concentration may be greater than in the entire sample volume [40]. It is worth noting that an additional center was created during the annealing of the material. It has been found in the literature that amorphous SiO_2_ nanoparticles show emissions with maxima around 400 and 460 nm [39]. As noted before, the additional band at about 450 nm correlates with the emission from the amorphous silica lattice. The literature identifies the SiO_2_ emission as the emission from the material’s surface (new luminescence center). It is probably possible due to the presence of silanol groups absorbed on the material’s surface [47]. It is also reported that the appearance of an additional emission band may be related to the long-term aging process of samples in the air [23]. This emission occurs while annealing at 100–400 °C. It often does not occur in materials that have not been subjected to thermal processes [48].

**Figure 4:**
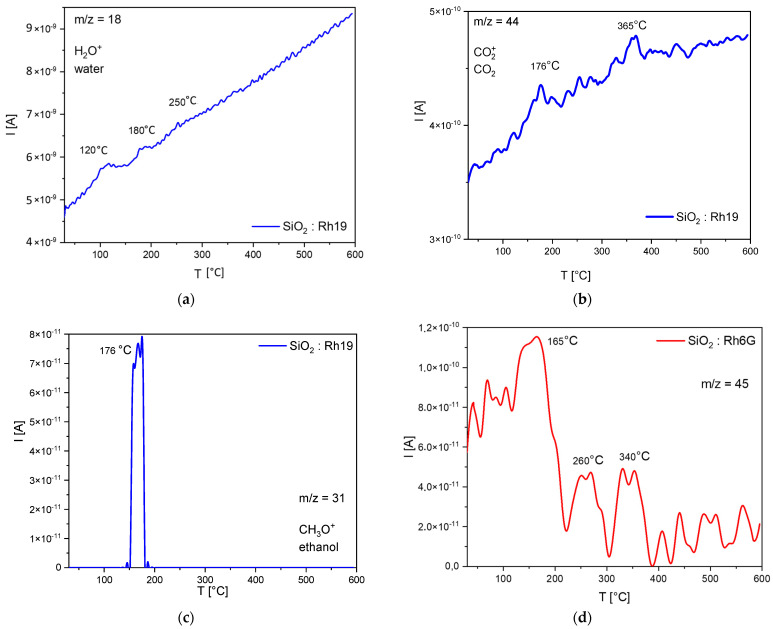
QMS−QMID (quadrupole mass analyzer-quasi multiple ion detection) analysis results for m/z (mass−to−charge ratio): (**a**) 18, (**b**) 44, (**c**) 31, (**d**) 45.

In order to test the hypothesis about the possible protection of the dye in the silica network and check whether it is possible to confirm the degradation of silanol groups (probably responsible for the emission at 450 nm), temperature tests were carried out. The thermal analysis shows that five stages of decomposition can be observed. The differences in individual mass loss values are shown in Table 2. By comparing individual mass loss values with the resulting gaseous products (QMS/FTIR), it is possible to estimate the degradation mechanism of the analyzed samples (Figure 4). The results of the TG analysis are plotted in Figure S8.

The first loss (30–50 °C) is related to the desorption of moisture from the sample—most likely water adsorbed on the surface. The next step (50–138 °C) concerns the desorption of the permanent moisture bonded to the material’s surface—chemically bonded moisture or a water condensation reaction in the sol-gel system. Further heating of the materials (138–200 °C) likely results in the alcohol condensation reaction in the sol-gel system, which produces primary alcohol (e.g., ethanol). The next step is related to the melting and partial thermal decomposition of rhodamine, which is most likely absorbed at the surface (200–300 °C), consistent with the data sheets of the dyes used. During this process, water and carbon dioxide are released. A further increase in temperature (above 450 °C) results in a progressive evolution of water and carbon dioxide (without a clear peak), most probably associated with the next step of rhodamine decomposition and/or further reactions of the sol-gel system. The maximum of this process is probably observed above the set temperature range.

The same degradation steps but different gaseous products are observed in the sample doped with Rh6G, where no water or ethanol is released as well as another compound based on carbon, oxygen, and hydrogen or nitrogen (m/z = 45: C_2_H_5_O^+^, CHO_2_^+^ or C_2_H_7_N^+^). Considering the FTIR (Supplementary Materials, Figures S6 and S7) results obtained for this sample, it is postulated that the decomposition product of this system does not include nitrogen-containing compounds or carboxyl groups (no corresponding peaks in the spectra). Based on the spectra with the literature data, it is suspected that they may be compounds of the following type: ethyl ether, 2-hydroxy derivatives, lactones, ethyl sulfonate, and ethylsulfones (C_2_H_5_O^+^) [49].

### 2.3. Chemical/Physical Etching Tests

The prepared structures were etched using an SI 500 reactive ion etching system manufactured by SENTECH Instruments. This system uses the reactive ion etching (RIE) method and has an additional inductively coupled plasma (ICP) source. The main advantage of the ICP-RIE etching method is the use, in one process, of a combination of physical etching by bombarding the sample with ions as well as chemical etching. The assumed goal of the technological research was to obtain smooth side walls of the waveguide. The produced side edges of the rib waveguide must have the lowest possible roughness to guarantee low optical losses; therefore, the etching quality of the side edge of the waveguides is also very important. Waveguides with organic dyes are one of the less-studied branches in photonics, especially in miniaturized integrated photonics [50,51,52].

**Figure 5:**
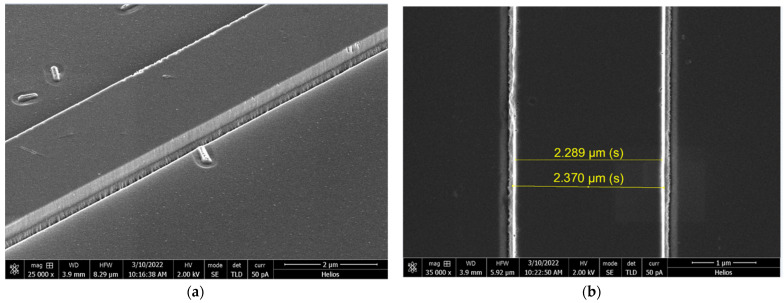
SEM images prepared for layer doped with Rhodamine **6G** after etching (CHF_3_-50, Ar-5, 0.3 Pa, 600 W, 165 V, oil, 26 °C, 200 s; the depth of etching was 350 nm): (**a**) etched layer, side view; (**b**) the etched layer, top view, and the marked scale correspond to the distance between the two boundaries of the waveguide.

Figure 5a shows the image of the edge of strip waveguides. Smooth side edges were obtained during etching. The depth of etching was 350 nm, and the sol-gel layer was around 200 nm thick. The photo clearly shows where the sol-gel layer ends, as the etched substrate is rougher [53].

## 3. Conclusions

In this study, we examined changes in the emission color of Rhodamine 19- and 6G-doped SiO_2_ upon annealing. The possible observed effects were analyzed based on measurements made for solutions, a thermal study using TG-QMS and available literature reports. Observations were made about concentration-dependent emissions in obtained materials. Despite similarities in dye structure, the synthesized material degradation mechanisms are different, while spectroscopic properties are practically analogous. During longer thermal processing at 300 °C, an additional band is visible, which can be attributed to SiO_2_ emission.

The presented method opens up new possibilities for the modification of silica, not only using different concentrations of dye but also emission color changes during annealing. The study showed that even upon annealing at 300 °C, the emissions of the organic dye are still visible. Materials doped with dyes from the same group as presented, synthesized as part of earlier research, showed no emissions upon annealing. Etching tests presented in this study show that it is possible to prepare active photonics components from synthesized sol-gels doped with organic dyes. The resulting side edges of the waveguide were smooth. In order to further investigate the functionality of the obtained waveguides, further tests are necessary, for example, for waveguides on a Si/SiO_2_ substrate.

## 4. Experimental Methods

Firstly, concentrated ethanolic solutions of dyes were prepared. These solutions were diluted with 96% ethanol to the desired concentration. Based on the available literature data, solutions were optimized to the concentration level of 0.01 mM in order to obtain mainly fluorescent dimers [13,14,54]. Mixtures of TEOS and 96% ethanolic solution of rhodamine were made in a 1:1.5 volume ratio in glass vials. A total of 0.1 volume of deionized water and 0.1 M HCl as catalyst were added. Glass vials were covered with parafilm and placed in a water bath sonicator for 3 h at 50 °C. Next, upon reaction, sols were left to dry in a fume cupboard for about a month at room temperature. Clear, transparent gels were obtained. Dry gels were put in a crucible and annealed to the maximum of 300 °C while the temperature increased by 2.5 °C/min. For etching tests, the sol was applied to the layers using dip coating, with a withdrawal speed of 180 mm/min. Upon gelation, samples started decreasing in volume due to the drying process. Since acid molecules do not dispose from the matrix as efficiently as water and solvent particles, pH rises [12]. This may indicate changes in spectroscopic properties. The decision was made to conduct thermal as well as spectroscopic analyses since all samples were still showing emissions upon excitation with a UV lamp.

Thermogravimetric analysis (TG) was carried out together with mass spectroscopy of gas products (QMS) and infrared spectroscopy (FTIR) with a STA 449 F1 Jupiter Netzsch thermal analyzer coupled with a QMS Aëlos 403D Netzsch quadrupole mass spectrometer and a Tensor 27 EQ Bruker spectrophotometer. Temperature measurements were performed within the range from 30 to 600 °C, with a heating rate of 10 °C/min and a nitrogen atmosphere (50 mL/min) in a corundum crucible with an insert and one hole in the lid. Absorption measurements were performed by an Evolution 300 Thermofisher spectrophotometer at room temperature in the range of 350–650 nm. Measurements of emission spectra were performed on an Edinburgh Instruments FLS980 spectrofluorometer, equipped with a xenon lamp with a measurement range from 230 to 1000 nm. Optical filters were used during the measurement of dried gels.

## Figures and Tables

**Table 1:** Concluded concentration in gels based on the references in solutions and literature.

Rhodamine 19	Emission Maximum (nm)					
	585	580	571	564	556	Organic dye solution used as reference
Concentration in gel	10^−3^ mol/L	5 × 10^−4^ mol/L	10^−4^ mol/L	5 × 10^−5^ mol/L	10^−6^ mol/L	[43,44]
**Rhodamine 6G**	**Emission maximum (nm)**					
	**588**	**577**	**570**	**551**	**Organic dye solution used as reference**	
Concentration in gel	5 × 10^−3^ mol/L	5 × 10^−4^ mol/L	10^−4^ mol/L	5 × 10^−7^ mol/L	[46]	

**Table 2:** Differences between individual mass loss values for annealed samples.

Dopant (Organic Dye)	Δm [%]					
	30–50 °C	50–138 °C	138–200 °C	200–450 °C	450–600 °C	∑ Δm
Rh6G	0.47	2.94	2.88	3.74	2.16	12.20
Rh19	0.66	2.81	2.51	4.55	2.11	12.62

## Data Availability

Data are included within the Supplementary Material.

## Supplementary Materials

The following supporting information can be downloaded at: https://www.mdpi.com/article/10.3390/gels8070408/s1. Figure S1. Illustrative scheme of sol-gel synthesis of gel doped with rhodamine dyes. Figure S2. Chromacity diagram for SiO_2_ doped with (a) Rh19 and (b) Rh6G after annealing. Figure S3. Absorption spectra recorded for different annealing temperatures for (a) SiO_2_:Rh19; (b) SiO_2_:Rh6G. Figure S4. Emission spectra recorded for different pH for (a) Rh19; (b) Rh6G. Figure S5. Emission spectra recorded for different concentrations for (a) Rh19; (b) Rh6G. Table S1. List of temperatures of the maximum rate of degradation. Figure S6. FTIR results of the SiO_2_: Rh6G sample: 100 °C, 170 °C, 240 °C, 350 °C, 518 °C. Figure S7. FTIR results of the SiO_2_: Rh19 sample: 100 °C, 170 °C, 225 °C, 545 °C. Figure S8. Results of thermogravimetric analyses: (a) mass loss graphs, (b) first derivative of mass loss graphs. Gel doped with Rhodamine 6G is marked green, while gel doped with Rhodamine 19 is marked red).
