# Supplementary material for: Concentration-Dependent Emission of Annealed Sol-Gel Layers Incorporated with Rhodamine 19 and 6G as the Route to Tunable High-Temperature Luminescent Materials
Source: Gels. 2022 Jun 28;8(7):408. doi: 10.3390/gels8070408 (PMC9319610; doi:10.3390/gels8070408)
Supplement: Supplementary file 1 [file gels-08-00408-s001.zip › gels-1771802-supplementary.pdf]

Supplementary Materials

Concentration dependent emission of sol-gel layers doped with Rhodamine 19 and 6G as the route to tunable luminescent materials

Maria Zdończyk, Bartłomiej Potaniec, Marta Fiedot-Toboła, Tomasz Baraniecki and Joanna Cybińska

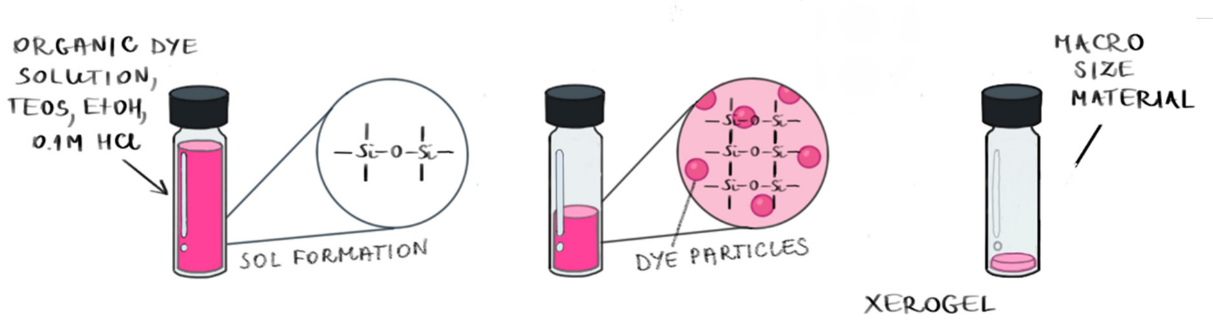

Figure S1. Illustrative scheme of sol-gel synthesis of gel doped with rhodamine dyes.

S1. Spectroscopic analysis

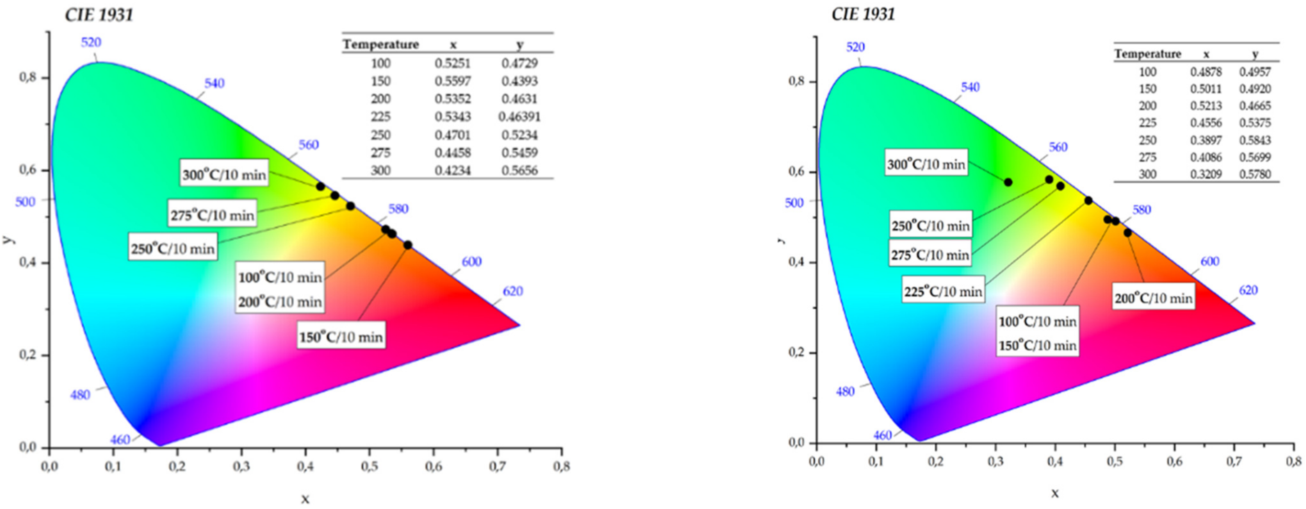

(a) **(b)**

Figure S2. Chromacity diagram for SiO_2 doped with **(a)** Rh19; **(b)** Rh6G, after annealing.

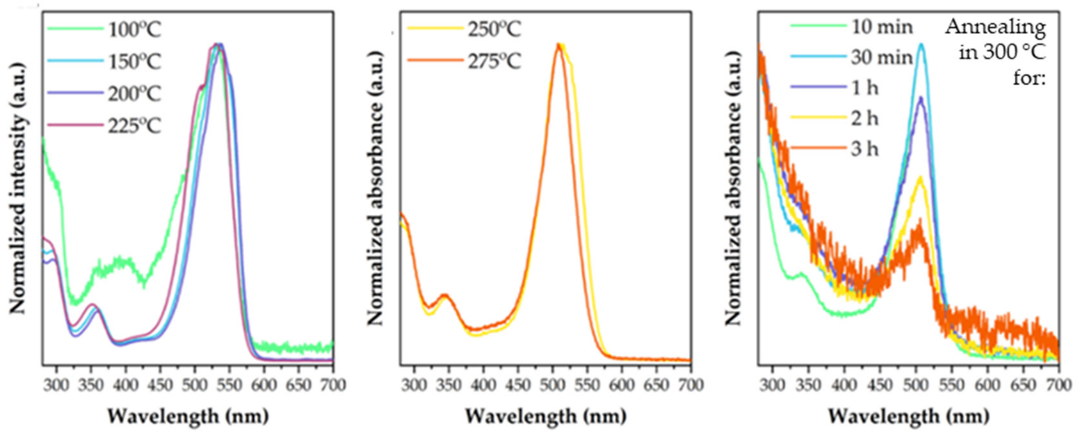

(a)

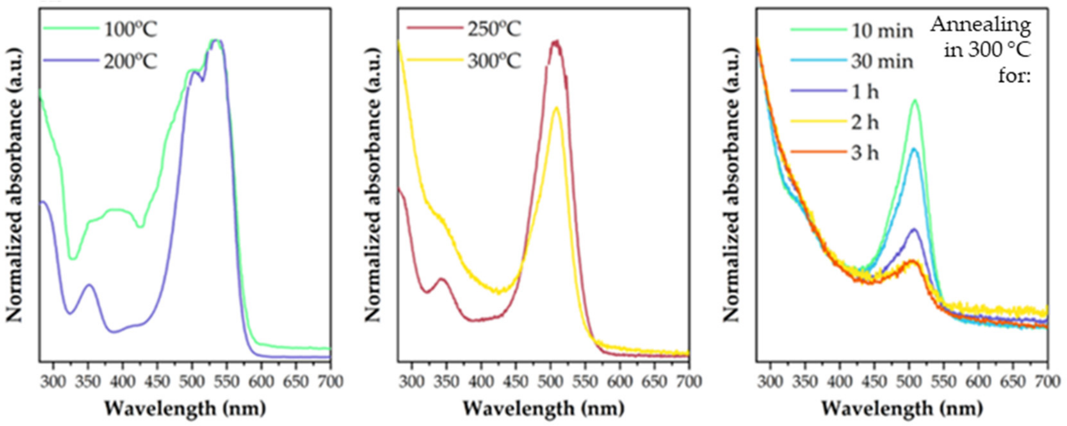

(b)

Figure S3. Absorption spectra recorded for different annealing temperatures for **(a)** SiO_2:Rh19; **(b)** SiO_2:Rh6G.

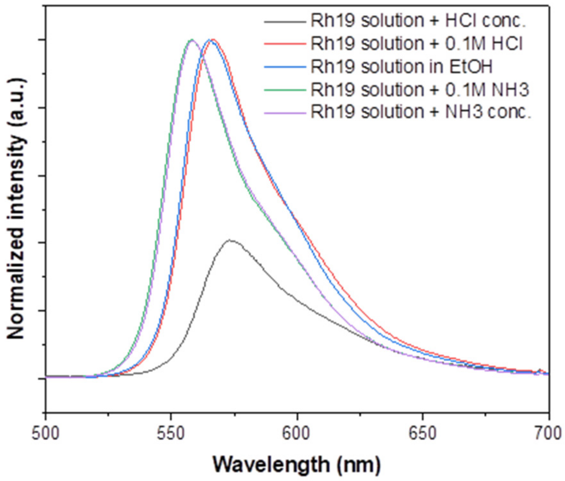

(a)

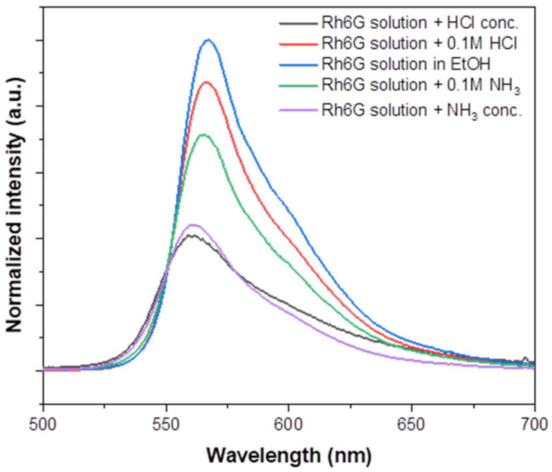

(b)

Figure S4. Emission spectra recorded for different pH for **(a)** Rh19; **(b)** Rh6G.

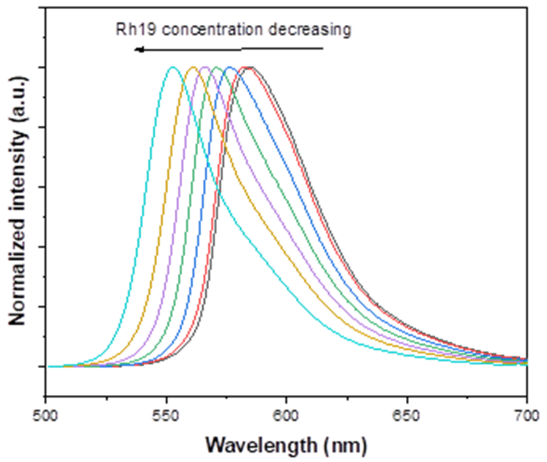

(a)

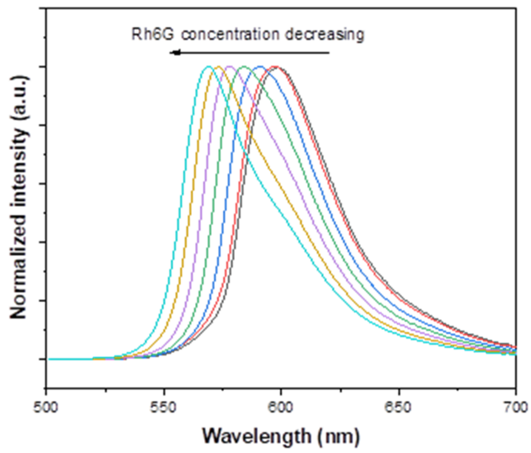

(b)

Figure S5. Emission spectra recorded for different concentrations for **(a)** Rh19; **(b)** Rh6G.

S2. Thermal analysis

Table S1. List of temperatures of the maximum rate of degradation.

Sample	DTG_1 [°C]	DTG_2 [°C]	DTG_3 [°C]	DTG_4 [°C]	DTG_5 [°C]
SiO_2 : Rh19	-	100	164	226	546
SiO_2 : Rh6G	-	100	166	245	514

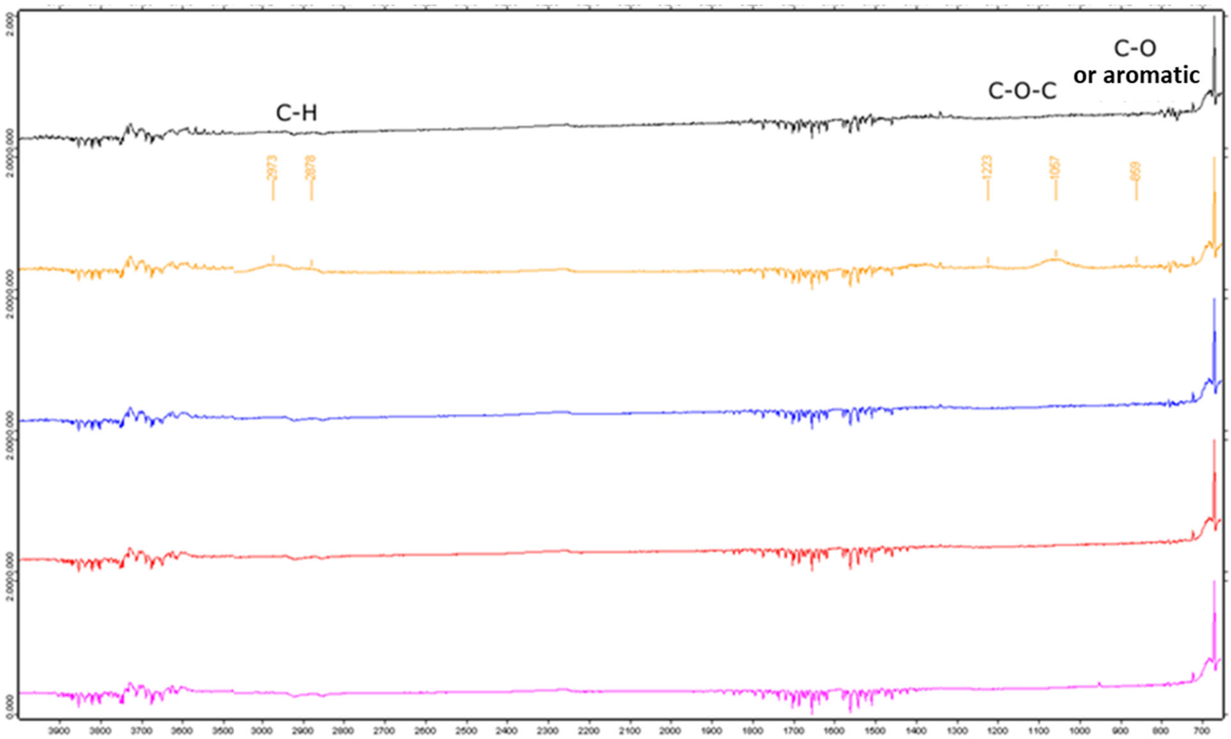

Figure S6. FTIR results of the SiO_2 : Rh6G sample: 100 °C, 170 °C, 240 °C, 350 °C, 518 °C.

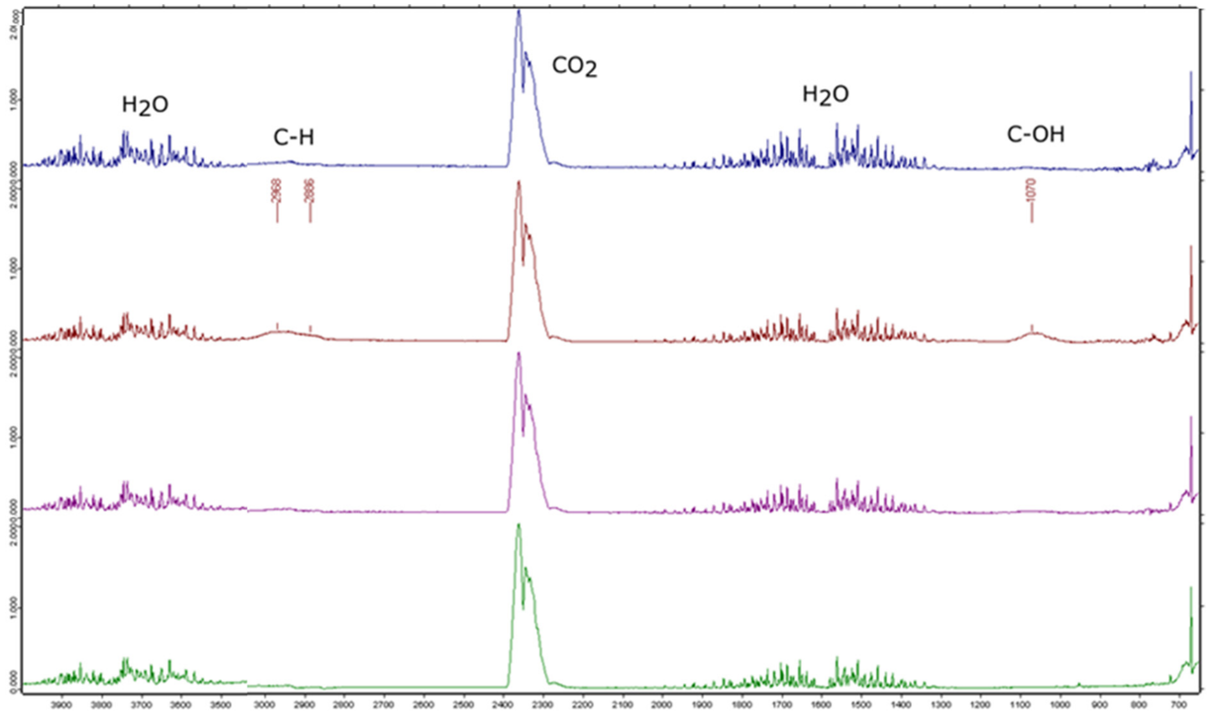

Figure S7. FTIR results of the SiO_2 : Rh19 sample: 100 °C, 170 °C, 225 °C, 545 °C.

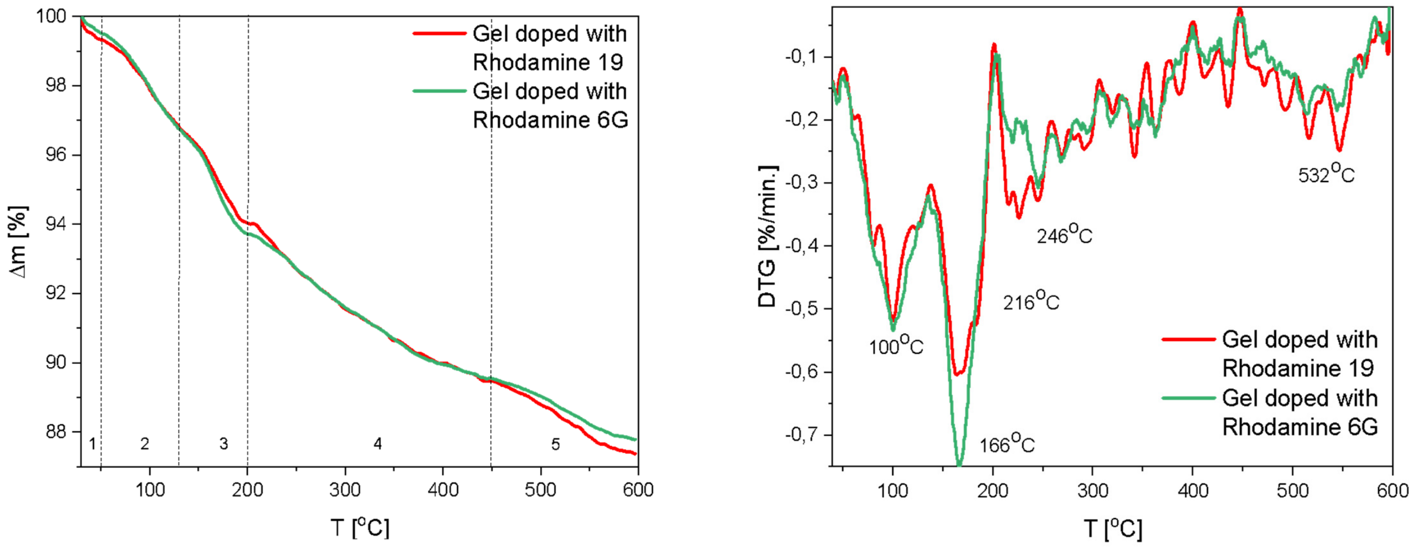

a) (b)

Figure S8. Results of thermogravimetric analyzes: (**a**) weight loss graphs, (**b**) first derivative of weight loss graphs. With green color gel doped with Rhodamine 6G is marked, while with red – Rhodamine 19.
